# Does External Knowledge Sourcing Enhance Market Performance? Evidence from the Korean Manufacturing Industry

**DOI:** 10.1371/journal.pone.0168676

**Published:** 2016-12-22

**Authors:** Kibaek Lee, Jaeheung Yoo, Munkee Choi, Hangjung Zo, Andrew P. Ciganek

**Affiliations:** 1 School of Business and Technology Management, College of Business, Korea Advanced Institute of Science and Technology (KAIST), Daejeon, Republic of Korea; 2 Korea Research Institute of Chemical Technology (KRICT), Daejeon, Republic of Korea; 3 Software Policy and Research Institute, Seongnam, Republic of Korea; 4 College of Business and Economics, University of Wisconsin-Whitewater, Whitewater, WI, United States of America; Universidad Veracruzana, MEXICO

## Abstract

Firms continuously search for external knowledge that can contribute to product innovation, which may ultimately increase market performance. The relationship between external knowledge sourcing and market performance is not well-documented. The extant literature primarily examines the causal relationship between external knowledge sources and product innovation performance or to identify factors which moderates the relationship between external knowledge sourcing and product innovation. Non-technological innovations, such as organization and marketing innovations, intervene in the process of external knowledge sourcing to product innovation to market performance but has not been extensively examined. This study addresses two research questions: does external knowledge sourcing lead to market performance and how does external knowledge sourcing interact with a firm’s different innovation activities to enhance market performance. This study proposes a comprehensive model to capture the causal mechanism from external knowledge sourcing to market performance. The research model was tested using survey data from manufacturing firms in South Korea and the results demonstrate a strong statistical relationship in the path of external knowledge sourcing (EKS) to product innovation performance (PIP) to market performance (MP). Organizational innovation is an antecedent to EKS while marketing innovation is a consequence of EKS, which significantly influences PIP and MP. The results imply that any potential EKS effort should also consider organizational innovations which may ultimately enhance market performance. Theoretical and practical implications are discussed as well as concluding remarks.

## Introduction

Open innovation, which is a method of collaboration that incorporates external knowledge sources, has received significant attention from both academia and industry [1]. The value of open innovation is that firms can overcome limitations in internal capabilities with external knowledge, which may generate innovative products in response to market changes [2,3]. Open innovation has become commonplace in many industries, including manufacturing and service industries, and has expanded as technology that facilities inter-organizational collaboration matures and competition increases.

Leading information technology (IT) companies such as Apple, Samsung, and IBM have established open business ecosystems to work with external partners, developers, consumers, and potential competitors. Apple’s success is an exemplary case of open innovation. Apple was able to increase its corporate value and competitiveness by seamlessly integrating thousands of external developers to their App Store, an online platform for mobile applications. Apple would have been challenged to replicate the same market performance had it relied solely on their own resources to develop the hundreds of thousands of applications available in their App Store [4].

The transaction cost of collaboration has dramatically diminished with external partners, such as customers, suppliers, universities, research institutes, and even competitors with the advancement in information and communication technologies (ICT). ICT allows firms to collect and utilize information from a much broader, diverse, and relevant segment of external partners than possible with traditional approaches to secure external knowledge like focus group interviews. Firms are increasingly adopting external knowledge from user innovations and crowdsourcing in their new product innovations [5,6]. Firms understand the importance of collaborating with external partners to better respond to changes in markets and explore new markets [7].

Research acknowledges the positive effect of external knowledge sourcing on product innovation [8–12]. Empirical studies on the causal mechanism from external knowledge sourcing to better market performance are far less common [13]. Firms anticipate profit from product innovation with investments in ICT to facilitate collaboration and knowledge exchange with external partners. However, not all resultant product innovations are successful. Numerous external (e.g., overall economic conditions, technological changes, social trends, political environments, etc.) and internal (e.g., each firm’s research and development capabilities, organization management, marketing, financial management, etc.) factors may explain market success [14]. Market success can be directly attributed to the innovations and strategies firms employ, assuming external factors influence all firms indiscriminately [15].

An examination of the innovations and strategies firms employ is necessary to better understand the causal mechanism from external knowledge sourcing to better market performance. Innovative firm activities that are non-technical in nature include organization and marketing innovation, but exclude product innovation [16]. A study of market performance for product innovation should account for a variety of innovative activities, including the firm’s external knowledge sourcing, product innovation, organization innovation, and marketing innovation [17,18]. Research has not extensively evaluated the role that such a variety of innovative activities has in the relationship between external knowledge sourcing and market performance. This study addresses this need by positing and empirically testing a theoretical model which links external knowledge sourcing to a variety of innovative firm activities.

The remainder of this manuscript is organized as follows. Section 2 of this paper reviews the theories that are the foundation of the research model. The research model and hypotheses are developed in Section 3. Section 4 presents the research methods. The results are presented in Section 5. Section 6 discusses the study results including the including the relevance that this research has for researchers and practitioners. Section 7 offers concluding remarks as well as study limitations, which reveal opportunities for future research.

## Literature Review

### External knowledge sourcing and product innovation

Firms pursue external knowledge sourcing to better respond to competitive environments [19]. Christensen argues that firms employing external knowledge sourcing should be able to capture new knowledge and technologies that can challenge leading companies [20]. Teece et al. [21] emphasize the importance of dynamic capabilities, like acquiring new knowledge and technologies to respond to a dynamic market, for a firm to be competitive. External knowledge sourcing is a cost-effective method of securing relevant knowledge and technologies in a contemporary environment characterized by short product life-cycles limited by closed in-house research and development [3].

Firms also pursue external knowledge sourcing to acquire knowledge and technologies for product innovation like new product releases [22,23]. Product innovation is better achieved accessing a diverse breadth of knowledge available from collaboration with different external actors like suppliers, research institutes, universities, customers, and even competitors [3,24]. The focus group interview, which collects new product information from a relatively limited subset of consumers, has changed significantly through online crowdsourcing. Online crowdsourcing platforms like ‘NineSigma’ and ‘InnoCentive’ publicly disclose their research and development problems online so that millions of minds across the world may solve them. Social media is increasingly leveraged to gauge consumer sentiment and solicit real-time feedback [4], which is timely knowledge that previously took weeks to capture in focus group interviews.

### External knowledge sourcing, firm’s innovation activities, and market performance

External knowledge sourcing may facilitate product innovation through collaborations with external partners. External knowledge alone does not guarantee market performance as firms require a variety of innovation activities (e.g., research and development, organization, marketing, etc.) to enhance market performance [17,25]. New knowledge may stimulate product innovation, a technical innovation, and refers to product performance and usage that is radically different or significantly improved [26]. Non-technical innovation, like organization and marketing innovation, supports product innovation [16]. Market performance, consequently, results from effective external knowledge acquisition and utilization as well as non-technological innovation [25]. The combination of technological and non-technological innovation activities is more effective to enhance and sustain market performance than technological innovation alone [27].

Previous research has focused on the relationship between external knowledge sourcing and product innovation. Kang and Kang [7] and Vega-Jurado et al. [24] analyzed the relationship between different knowledge sources and product innovation. Hwang and Lee [28] analyzed how the breadth or depth of external knowledge impacts product innovation. Tsai [8] examined the relationship between collaboration networks and product innovation performance employing absorptive capacity as a moderator. Studies of external knowledge sourcing have focused on the relationship between various knowledge sources and innovation performance, the breadth or depth of knowledge and innovation performance, and the moderating effect of absorptive capacity [29].

**Fig 1:**
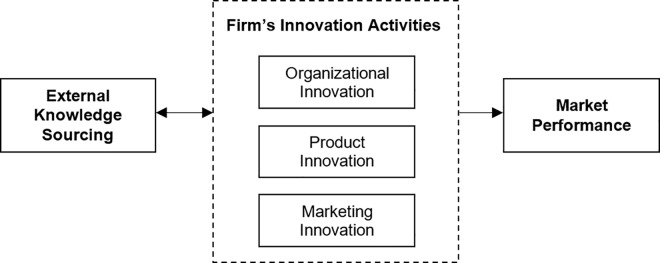
Study conceptual framework

Research has not fully examined the role that a firm’s non-technological innovations have on exploiting external knowledge sourcing for product innovation. Most firms plan for how to best utilize external knowledge before engaging in these initiatives. Firms rearrange internal processes, rules, and structures to maximize the benefits of external knowledge sourcing [30]. Non-technological innovation merits further examination to better explain the relationship between external knowledge sourcing and product innovation. Both technological and non-technological innovations influence product innovation, which impacts market performance. Fig 1 illustrates the study’s conceptual framework which proposes causal mechanism pathway from external knowledge sourcing to market performance.

## Research Model

### Product Innovation

#### External knowledge sourcing and product innovation performance

Firms engage in external knowledge sourcing to better respond to changing and dynamic markets [3,31]. Kang and Kang [7] found that manufacturing firms which participate in external knowledge sourcing collaborate with external partners on research and development and information transfer. Collaboration with suppliers may increase the quality of new products by co-generating ideas and solutions for product design [8]. Collaboration with external partners is also effective for solving short-term technical problems [9]. Collaboration with customers helps to identify market opportunities and improves product design during the early stages of technological development [10]. Competitors experience synergistic effects for solving common problems when they share technological knowledge and skills with each other [11]. Collaborations with competitors may also reduce the time and overall risk in technological development [12]. Collaboration with universities or public research institutes, which are sources of novel technologies and knowledge, is believed to greatly increase a firms’ research and development capabilities [12].

Global manufacturers commonly search for new products or ideas for product improvements though crowdsourcing or other collaboration programs [4]. IBM prioritizes the suggestions they receive from customers to determine scheduling for research and development projects. Dell hosts an online community where customers engage and offer new product suggestions. MacPherson [32] found that external knowledge sourcing has a significant impact on product innovation performance. Ransley and Rogers [33] report that technologies transferred from external partners have a crucial role in the success of internal research and development efforts. This study posits a positive relationship between external knowledge sourcing and product innovation performance.

**H1.** External knowledge sourcing will positively affect product innovation market performance.

#### Product innovation performance and market performance

Product innovation refers to the development and release of goods or services based on customers’ needs or market demands [34]. Product innovation includes both technology and market-related aspects [35] and is essential for market success given changing customer preferences and frequent shifts in technological development [36]. Product innovation may involve differentiation in both product quality and functionality, which should attract more customers. Product innovation increases a firm’s competitiveness, which boosts market position and enhances market performance [37]. This study posits a positive relationship between product innovation performance and market performance.

**H2.** Product innovation performance will positively affect product innovation market performance.

### Organizational Innovation

#### Organizational innovation and external knowledge sourcing

Becker and Dietz [18] argue that organization innovation is a prerequisite for external knowledge sourcing success as it entails coordinating, managing, and controlling cooperative activities with external partners. Trist and Bamforth [38] state that organization innovation may also include redesigning and restructuring an organization’s social system. Organization innovation may also involve the reallocation of roles and relationships between members of the organization as well as rearrangements in business procedures and structure [39]. Organization innovation can enhance a firm’s productivity if successful or foster disorder and inefficiency when challenged.

Firms do implement organization innovation when pursuing external knowledge sourcing initiatives. P&G, IBM, Apple, and GE are each Fortune 100 firms with departments that exclusively coordinate external knowledge sourcing. Siegel et al. [40] report that universities invest in technology licensing offices and technology transfer offices in pursuit of opportunities to collaborate with external partners. Firms also develop their own knowledge management systems to facilitate information exchanges with internal stakeholders and external partners. A proper and timely sharing of relevant information should increase problem solving efficiency [41]. Some firms use online crowdsourcing to generate ideas from a broad, diverse, and relevant audience [4]. Organization innovation is essential to effectively use external knowledge [16]. This study posits a positive relationship between organization innovation and external knowledge sourcing.

**H3.** Organizational innovation will positively affect external knowledge sourcing.

#### Organizational innovation and product innovation performance

Internal research and development capability is commonly associated with product innovation implementation [24]. Internal research and development capability refers to a firm’s ability to integrate research and development strategy, project implementation, project portfolio management, and expenditures [42]. This capability is reinforced with organization innovation in leadership, talent management, knowledge management, and creativity management [43].

Many firms enhance product innovation performance through organization innovation. Firms employ a variety of organization innovation approaches as needed, like the creation of task force teams, restructuring, and human resource reallocation [44]. Firms adopt information systems to increase internal communication and business process efficiency, which ultimately enhances product innovation [45]. This study posits a positive relationship between organization innovation and product innovation performance.

**H4.** Organizational innovation will positively affect product innovation performance.

#### Organizational innovation and market performance

Firms employ organization innovation to enhance market performance [46]. Firms commonly develop strategic alliances with external partners to increase market share or expand sales [27]. Damanpour and Evan [27] caution that organization innovation involves both visible and invisible costs for the firm. Firms encounter transition costs associated with restructuring and retraining implementing organization innovation. Cultural conflicts to a newly imposed system, business inefficiencies, and member complaints are additional costs associated with organization innovation [47]. Becker and Dietz [18] state that a proper alignment of external resources with a firm’s technological capabilities along with an appropriate adjustment of a firm’s organizational structure to exploit external innovation may contribute to a firm’s market performance. This study posits a positive relationship between organization innovation and market performance.

**H5.** Organization innovation will positively affect market performance.

### Marketing Innovation

#### Marketing innovation and external knowledge sourcing

Marketing innovation is undergoing a paradigm shift with influence historically originating from internal experts now shifting to consumers [22]. Consumers have greater influence now using online social networking than ever before on nearly the entire product production process, including design, initial word-of-mouth, and diffusion [48]. The emergence of empowered users motivates firms to collaborate with external partners like research institutes, customers, and even competitors to better respond to diverse consumer demands for innovative products [49].

Firms seek external knowledge from diverse sources as products become more complex [4]. Firms are challenged to develop innovative products in a dynamic market relying only upon their internal resources. External knowledge sourcing supports marketing innovation by improving the accuracy and efficiency of identifying consumer demands, leading to offering the ‘right’ product at the right place, price, and time [50]. This study posits a positive relationship between external knowledge sourcing and marketing innovation.

**H6.** External knowledge sourcing will positively affect marketing innovation.

#### Marketing innovation and product innovation performance

Marketing innovation includes tasks to better understand consumer needs, pioneering new markets, and product targeting to maximize sales profits. Firms invest significant resources in market surveys to identify consumer demands and needs to better understand what to provide [51] and not depend solely upon the assessments of internal experts [52]. A more accurate and objective market evaluation market is possible when the opinions of a variety of stakeholders, including external partners, are incorporated in the product innovation process.

External perspectives can generate ideas for new products. User innovation is one way to integrate users in the product innovation process [5]. Market opinion leaders and new product early adopters are more easily accessible and activated with online services. Firms can also hold idea contests as well as directly engage users in discussion forums to capture new ideas. External knowledge sourcing can generate insights for marketing innovation that may lead to product innovation.

External experts and suppliers are equally important as consumers in the product innovation process. Firms must monitor the emergence of ‘disruptive innovation’ Christensen [20] or risk falling behind in product innovation (e.g., Kodak, Nokia, Motorola, etc.). Firms eagerly seek information on competitor's products and leverage that information for advantage by reflecting upon their own product innovation process. This study posits a positive relationship between marketing innovation and product innovation performance.

**H7.** Marketing innovation will positively affect product innovation performance.

#### Marketing innovation and market performance

Marketing innovation derived from external knowledge sourcing influences product innovation, including the decision-making process of designing and planning the ‘right’ product [5,22]. External knowledge sourcing may also reveal information about product prices, distribution, and public relations as well as technological and functional specifications. Firms can obtain information from external partners about the ‘right’ time, place, and price along with knowledge of what kind of products should be developed [53].

Product innovation can experience challenges, including what Moore [54] described as ‘Chasm’ and ‘Death Valley’. Rogers [55] theory of diffusion argues that product innovation must consider functional features, like relative advantage and compatibility, in addition to minimizing access barriers, like price and sales locations. Product innovation should also be publicized to a target of leading consumers. Firms utilize social media to encourage market opinion leaders and new product early adopters to promote product innovation through word-of-mouth. Firms often plan and design product innovation targeting leading consumer needs or offer chances to experience the product early in the product production process [56]. This study posits a positive relationship between marketing innovation and market performance.

**H8.** Marketing innovation will positively affect market performance.

### Research Model

**Fig 2:**
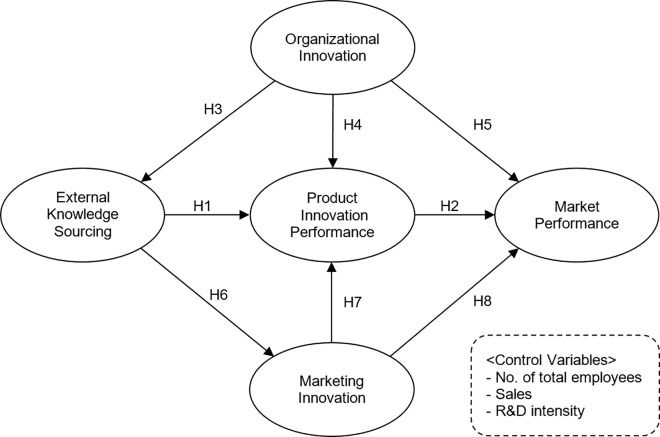
Proposed research model

Fig 2 illustrates the study’s research model, which captures the causal mechanism from external knowledge sourcing to market performance. The proposed research model addresses the following research questions: does external knowledge sourcing lead to market performance and how does external knowledge sourcing interact with a firm’s different innovation activities to enhance market performance. Control variables are employed to control the potential for bias from confounding effects. The study control variables include the number of total employees, sales per the number of total employees, and research and development intensity per the number of total employees.

## Methodology

### Data and analysis method

Data was collected from the 2014 Korean Innovation Survey (KIS) to validate the proposed research model. The KIS is a nationwide survey, recurring every 2 to 3 years, which addresses the innovation activities and financial results for all registered firms. The questions in the KIS are based on the Oslo Manual, third edition (see OECD [16]), and the 2012 European Community Innovation Survey. The KIS is comprehensive as the survey includes direct measures of innovation and financial performance along with a wide variety of factors that influence innovation.

The study’s target population is Korean manufacturing companies established before 2011 with over 10 employees. 46,101 companies satisfied these criteria in the KIS Database. 4,031 firms which met the study criteria were selected for analysis using a stratified sampling. Firms that reported no innovation activities within the past three years were excluded from the sampling in addition to responses with missing or erroneous data, resulting in a final sample of 1,059 manufacturers. The firms included in the final sampling are categorized into 23 industries (see Table 1).

**Table 1:** Study sampling

KSIC*	Industry [Manufacturing sector]	A population		Selected samples	
10	Food	2,524	5.5%	66	6.2%
11	Beverages	151	0.3%	6	0.6%
13	Textile	2,287	5.0%	25	2.4%
14	Wearing apparel, fur	1,236	2.7%	11	1.0%
15	Leather, shoes	436	0.9%	6	0.6%
16	Wood	581	1.3%	5	0.5%
17	Pulp, paper	1,159	2.5%	13	1.2%
18	Printing, paper press	913	2.0%	8	0.8%
19	Coke, petroleum refining	114	0.2%	3	0.3%
20	Chemical compounds	1,893	4.1%	82	7.7%
21	medicine and medical supplies	352	0.8%	36	3.4%
22	Plastic, rubber	3,985	8.6%	81	7.6%
23	Non-metallic minerals	1,791	3.9%	30	2.8%
24	Basic metals	2,090	4.5%	20	1.9%
25	Fabricated metals	6,356	13.8%	113	10.7%
26	Electronic components, telecommunication	3,196	6.9%	152	14.4%
27	Medical, precision machinery	1,629	3.5%	56	5.3%
28	Electrical machines	3,023	6.6%	95	9.0%
29	Machinery	6,924	15.0%	138	13.0%
30	Automobiles	2,893	6.3%	73	6.9%
31	Transportation equipment	973	2.1%	12	1.1%
32	Furniture	836	1.8%	17	1.6%
33	Others	759	1.6%	11	1.0%
**Total**		46,101	100.0%	1,059	100.0%

*KSIC: Korea Standard Industry Code 9

Partial least squares (PLS) was used to analyze the data sampling. PLS is accepted as an appropriate statistical model for structural path analysis [57] and allows the testing of hypotheses with formative latent variables [58]. PLS is an appropriate method for this study since the research model includes structural paths with formative latent variables (i.e., external knowledge sourcing, organization innovation, and marketing innovation). The data analysis was performed using Smart PLS (version 2.0.M3).

### Variables and measures

The research model includes reflective and formative constructs. Reflective measurement theory is based on the assumption that latent constructs cause the measured variables while measurement error results in an inability to fully explain these measures. Formative measurement theory assumes that the measured variables cause the construct while measurement errors are the inability to fully explain the construct [58]. Reflective items are representative of the same conceptual domain while formative items define the construct. External knowledge sourcing, organization innovation and marketing innovation constitute formative constructs in the research model while product innovation performance and market performance are reflective constructs.

#### External knowledge sourcing (EKS)

Seven dimensions of EKS were utilized to construct a seven-item formative scale based upon previous research [8,11,59,60]. Survey respondents were asked if external knowledge sourcing is used for any innovation activities within the past three years. If the survey respondent indicated that innovation activities were used, the respondent was asked to evaluate the importance of the activities. Each item was rated on a four-point scale ranging from ‘0’ (“none”) to ‘3’ (“strongly agree”).

#### Organizational innovation (OGI)

OGI was measured using a three-item formative scale, following the Oslo Manual, third edition. Survey respondents were asked whether their firm introduces and implements organizational innovation in following areas: business practices, workplace organization, and external relations [16]. Each item is a binary variable, coded ‘1’ if the firm introduces and/or implements such an activity and ‘0’ otherwise.

Business practices include initiating new methods for organizing routines and procedures to conduct work. One type of business practice involves implementing new management systems, such as supply chain management, six sigma, knowledge management, business process re-engineering, quality management, and education/training. Knowledge management includes adopting new practices to improve organizational learning and knowledge sharing. Workplace organization includes initiating new methods for delegating responsibility and decision making among employees, but also includes the integration of new business activities. External relations involve fostering new ways of organizing relations with external organizations. Examples of external relations include establishing new collaborations with research organizations or customers, new methods of integration with suppliers, and outsourcing organizational activities.

#### Marketing innovation (MKI)

MKI was measured using a four-item formative scale, based on the Oslo Manual, third edition. Survey respondents were asked whether their firm introduces and implements marketing innovations in areas like design and packaging, promotion, placement, and pricing [16]. Each item is a binary variable, coded ‘1’ if the firm introduces and/or implements such an activity and ‘0’ otherwise.

Design and packaging includes changes that are intended to enhance product appeal or to target a new market or market segment. Promotion includes promotional efforts made by firms to improve their product's image or to increase product awareness. Placement includes both the channels that firms select to sell their products and also how those channels are designed to best market their products. Price involves the use of pricing methods to market goods or services.

#### Product innovation performance (PIP)

Prajogo and Ahmed [43] designed a construct for measuring PIP based on criteria that was conceptualized in previous innovation studies (e.g., Deshpandé et al. [61]). The PIP criteria include the level of newness of new products, the speed of new product development, the number of new products introduced to the market, and the number of new products that are first-to-market. PIP was measured using a four-item reflective scale. Survey respondents were asked about the degree of various outcomes for the variety of products, replacement of old products, early market entrants, and quality enhancement of products. Survey respondents rated all items on a four-point scale ranging from ‘0’ (“none”) to ‘3’ (“strongly agree”).

#### Market Performance (MP)

MP was captured with marketable outputs of innovative products like revenue, ratio of new product sales, and new product success rate [62]. Link and Scott [63] operationalize MP as innovative sales productivity, which is the ratio of sales attributed to new products divided by the total number of employees. MP is measured in this study using innovative products’ sales ratio and innovative products’ sales per employee. Table 2 summarizes the measurements employed in this study along with relevant studies that support the use of these measurements.

**Table 2:** Study variables and measurements

Constructs	Indicators	References
External Knowledge Sourcing (EKS)	Suppliers	[8], [11], [59], [60]
	Customers (public sector)	
	Customers (private sector)	
	Competitors (or other firms)	
	Private services (consulting)	
	Universities	
	Public R&D institutions	
Organizational Innovation (OGI)	Business practices	[16]
	Workplace organization	
	External relations	
Marketing Innovation (MKI)	Design and packaging	[16]
	Promotion	
	Placement	
	Pricing	
Production Innovation Performance (PIP)	Variety of products	[43], [61]
	Replacement of old products	
	Early market entrants	
	Quality enhancement of products	
Market Performance (MP)	Innovative products’ sales ratio	[62], [63]
	Innovative products’ sales per employee	

#### Control variables

Firm size and research and development intensity are controlled to account for potentially confounding effects. Firm size affects research and development strategies and performance because larger firms typically have more resources to devote to customer relationship management, marketing research, research and development, and networking activities [64]. Small firms typically outperform larger and more established counterparts in terms of creativity, speed, and flexibility [65]. The number of employees and sales per employees are controlled, which represents the ratio of total sales of the firm divided by the total number of employees. Internal research and development efforts influence the effectiveness of innovation strategies in studies that examined the effect of inter-organizational collaboration on innovation performance [66]. Research and development intensity is also controlled, which represents the ratio of internal research and development expenditures divided by the total number of employees.

## Results

### Measurement model

A confirmatory factor analysis (CFA) was conducted to test the measurement model. The convergent and discriminant validity of the constructs were examined to validate the measures employed. The composite reliability (CR) for each scale was calculated to analyze the internal consistency of the latent variables. Reliability coefficients of 0.70 or higher are generally considered adequate [67]. The CR values of all reflective constructs (i.e., product innovation performance and market performance) were above 0.70 (see Table 3).

**Table 3:** Factor loadings and AVE of latent variables

Constructs	Indicators	Loadings	AVE	Composite Reliability
External Knowledge Sourcing (EKS)	Suppliers	na	na	na
	Customers (public sector)			
	Customer (private sector)			
	Competitors			
	Private services (consulting)			
	Universities			
	Public R&D institutions			
Organizational Innovation (OGI)	Business practices	na	na	na
	Workplace organization			
	External relations			
Marketing Innovation (MKI)	Design and packaging	na	na	na
	Promotion			
	Placement			
	Pricing			
Production Innovation Performance (PIP)	Variety of products	0.713***	0.540	0.824
	Replacement of old products	0.766***		
	Early market entrants	0.792***		
	Quality enhancement of products	0.662***		
Market Performance (MP)	Innovative products’ sales ratio	0.825***	0.830	0.907
	Innovative products’ sales per employee	0.990***		

na. Loadings, AVE, and Composite Reliability are not applicable to formative constructs

***p < .01

Convergent validity is assessed by examining both factor loadings and the average variance extracted (AVE). The factor loading for each latent construct item was significant at the 0.01 level (see Table 3). AVE measures the overall proportion of variance accounted for in each latent construct item. Convergent validity was exhibited for each latent construct item as all shared variances were well above the recommended threshold level of 50% [67] (see Table 3). Discriminant validity was exhibited for each measure using item loadings, cross-loadings, the square root of the AVE, and a correlation matrix (see Tables 4 and 5). The CFA results support the reliability and validity of each measure.

**Table 4:** Discriminant validity (cross-loadings)

Indicators	EKS	OGI	MKI	PIP	MP
Suppliers	**0.646**	0.218	0.134	0.127	-0.011
Customers [public sector]	**0.569**	0.156	0.091	0.197	-0.030
Customers [private sector]	**0.505**	0.160	0.144	0.076	0.041
Competitors	**0.640**	0.215	0.125	0.135	0.017
Private services	**0.638**	0.226	0.179	0.061	0.032
Universities	**0.727**	0.241	0.144	0.157	-0.011
Public R&D institutions	**0.612**	0.210	0.120	0.121	-0.034
Business practices	0.282	**0.816**	0.384	0.232	-0.027
Workplace organization	0.265	**0.788**	0.371	0.225	-0.083
External relations	0.268	**0.848**	0.441	0.272	0.003
Design and packaging	0.189	0.405	**0.866**	0.318	0.040
Promotion	0.126	0.399	**0.704**	0.274	0.035
Placement	0.148	0.413	**0.780**	0.302	0.008
Pricing	0.182	0.417	**0.848**	0.314	0.030
Variety of products	0.129	0.164	0.262	**0.713**	-0.006
Replacement of old products	0.157	0.219	0.252	**0.766**	-0.011
Early market entrants	0.173	0.271	0.329	**0.792**	0.060
Quality enhancement of products	0.139	0.217	0.244	**0.662**	0.082
Innovative products’ sales ratio	0.086	0.068	0.112	0.148	**0.825**
Innovative products’ sales per employee	-0.024	-0.060	0.014	0.016	**0.990**

**Table 5:** Correlations of latent variables

Construct	EKS	OGI	MKI	PIP	MP
EKS	na				
OGI	0.331	na			
MKI	0.210	0.492	na		
PIP	0.205	0.300	0.373	*0*.*735*	
MP	-0.002	-0.035	0.036	0.044	*0*.*911*

Diagonal elements in italic font style are the square root of the AVE

na. AVE is not applicable to formative constructs

#### Hypotheses testing

**Fig 3:**
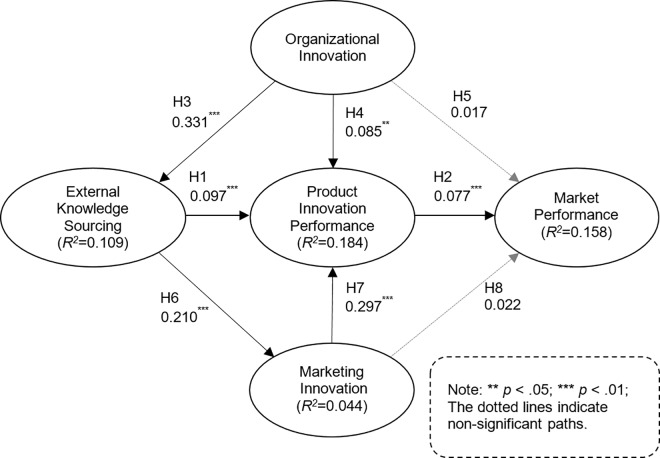
Hypotheses tests results

The structural equation modeling results are presented in Fig 3. The proposed research model hypotheses tests are summarized in Table 6. Six out of the eight hypotheses were supported in the data analysis. A bootstrapping re-sampling technique was employed to calculate the corresponding t-values for each hypothesized relationship.

**Table 6:** Summary of hypotheses testing results

Hypothesis		Path coefficient	t-value	Outcome
H1:	EKS → PIP	0.097	2.825***	Supported
H2:	PIP → MP	0.077	2.658***	Supported
H3:	OGI → EKS	0.331	9.955***	Supported
H4:	OGI → PIP	0.085	2.506**	Supported
H5:	OGI → MP	0.017	0.542	Rejected
H6:	EKS → MKI	0.210	5.688***	Supported
H7:	MKI → PIP	0.297	10.047***	Supported
H8:	MKI → MP	0.022	0.739	Rejected

**p < .05

***p < .01

The data analysis results support H1. External knowledge sourcing positively influences product innovation performance. The relationship between product innovation performance and market performance was also significant, which supports H2. The KPM chain (external knowledge sourcing → product innovation performance → marketing innovation) was statistically significant.

The data analysis results support H3. Organization innovation positively influences external knowledge sourcing. The relationship between organization innovation and product innovation performance was also significant, which supports H4. The data analysis results do not support H5 as the relationship between organization innovation and market performance was not statistically significant.

The data analysis results support H6. External knowledge sourcing positively influences marketing innovation. The relationship between marketing innovation and product innovation performance was also significant, which supports H7. The data analysis results do not support H8 as the relationship between marketing innovation and market performance was not statistically significant.

The data analysis results demonstrate that external knowledge sourcing has a significant and positive influence on market performance. The data analysis results also reveal a statistically significant causal path from organization innovation, through external knowledge sourcing, to marketing innovation.

## Discussions

This study provides empirical evidence of the chain effect from external knowledge sourcing (EKS) through product innovation performance (PIP) to market performance (MP). This chain effect confirms the positive influence of external knowledge sourcing on product innovation market performance. The extant literature primarily examines the causal relationship between external knowledge sources and product innovation performance while empirical analyses examining the influence on market performance is needed [13,45]. This study demonstrates that manufacturers intensify collaboration with external partners, which leads to product innovation and market sustainability [47].

The study results confirm that organization innovation influences external knowledge sourcing success. Research provides conflicting evidence regarding the direct/indirect relationship between external knowledge sourcing and organization innovation [18]. External knowledge sourcing generally has a positive influence on product innovation but doubts exist. Significant transaction and other associated collaboration costs engaging with external partners may exist (e.g., human resource, training, etc.) and challenge external knowledge sourcing initiatives [15]. The study results demonstrate that organization innovation significantly influences external knowledge sourcing success (β = 0.331). Moreover, the study results indicate that organization innovation enhances product innovation performance (β = 0.085). Organization innovation is an important and necessary condition of external knowledge sourcing success.

External knowledge sourcing success is maximized when accompanied by marketing innovation. External knowledge sourcing significantly influences marketing innovation (β = 0.210). The study results indicate that marketing innovation alone enhances product innovation performance (β = 0.297). Marketing innovation also indirectly enhances market performance through product innovation performance (β = 0.023 or 0.297 * 0.077). Marketing innovation success requires an understanding of broader and non-functional product features (e.g., prices, distribution, promotion, service ideas, etc.), which external knowledge sourcing facilitates. Marketing innovation is undergoing a paradigm shift with influence historically originating from internal experts now shifting to consumers [22]. External knowledge sourcing enables this shift from internal experts to a variety of external partners.

Organization innovation is an important prerequisite for collaboration with external partners. External knowledge sourcing requires top management support and an appropriate environment to enhance product innovation performance. Top management should encourage the use of collaborative technologies which facilitate exchanges with external partners, but also publically support external knowledge sharing within the firm. Top management can publically recognize successful product innovation resulting from external knowledge sharing, sponsor idea contests to stimulate external knowledge generation, and integrate new metrics for external knowledge sharing in performance evaluations (i.e., number of knowledge contributions which lead directly to product innovation and/or measureable results). For example, IBM and 3M allocate a significant portion of employee working time towards sharing knowledge. Top management must also adapt to the paradigm shift of power from internal experts to external partners, which may be a significant cultural shift to overcome.

An organization restructuring or consolidation of resources and tools may be necessary for a firm to support effective external knowledge sharing. These organization changes may lead to new core abilities [68] and enhance product innovation [28,69]. For instance, IBM created an internal forum for innovation named ‘Think Place’ for employees to share ideas and evaluate colleague work, which enhanced internal communication and idea development. IBM also runs ‘Insight Phase’, an independent conference system that furthers discussion of select ideas from its open innovation programs. Senior staff at the executive level participate in the conference system and apply ideas to commercialization. Google operates a ‘Peer Review’ system where employees review each other’s source code while Google ‘Moderator’ is designed to share information about internal resources so that employees can find relevant expertise. P&G supports a separate department dedicated to open innovation named C&D (Connect & Develop) and a C&D leader is incorporated into teach operational division. GE has a knowledge based innovation program named ‘Imaginational Breakthroughs’ to create and support new business models. Each of these Fortune 100 firms offer examples where organization restructuring or a shifting of resources occurred to facilitate knowledge sharing and enhance new product innovation.

Total effect size is the sum of the direct and indirect effects of the exogenous variable on the outcome. Accounting for the total effect size, PIP is the most salient factor affecting MP (0.077), followed by MKI (0.045), OGI (0.029), and EKS (0.017). Market Performance is better attained by a firm’s ability to identify the right time, place, and price to introduce new products to market (PIP) than an ability to implement organizational (OGI) or marketing innovations (MKI).

A statistically significant relationship did not exist between OGI and MP which might be due to confounding effects. Positive aspects of OGI on MP were considered in this study, such as reconfiguring business portfolios, enforcing marketing & sales divisions, and developing strategic alliances with external partners. Firms incur both quantitative and qualitative costs engaging in OGI, which may be difficult to accurately quantify. Transition costs result from restructuring and retraining organizational structures. Cultural conflicts, business inefficiencies, and member resistance can have a negative impact on firm performance [47]. Market performance should improve when OGI is effectively implemented. Challenges implementing OGI may adversely affect market performance, which is consistent with the findings of Damanpour and Evan [27].

The study results indicate that the primary role of organizational innovation should be to improve product innovation performance rather than to increase market performance. Strategic alliances or collaborations with external partners to seek better ideas and breakthrough technologies will lead to a greater chance of market success. Organizational support for open innovations that lead to idea generation and research are other possible ways to attain better market performance. Conflict management is essential to mitigate potentially negative organizational management side effects [70]. Intangible costs such as innovation resistance and employees’ dissatisfaction resulting from cultural change should be monitored and controlled [71]. Strong leadership from top management and peer-group support may better facilitate change with education and good communication practices [72].

MKI is an endeavor to raise the sales of innovative products through changes in product design, promotion, placement, and price. A statistically significant relationship did not exist between MKI and MP and was unexpected. Inappropriate or ineffective MKI implementation may lead to excessive costs and relatively poor performance. For example, an inappropriate price strategy or an improper differentiation of target customers may lead to lackluster demand and unsatisfactory market performance [54,55]. A statistically significant indirect effect of MKI on MP through PIP does exists. Firms must develop their monitoring capabilities to better recognize market trends, competitor capabilities, and customer sentiment. Firms must also foster impactful promotion programs with the launch of innovative products which effectively influence the experience of lead users and early adopters [73].

## Conclusions

Firms tend to have an unfriendly view towards knowledge and technologies acquired outside their organization. A ‘not-invented-here’ attitude harms competitiveness in dynamic markets which is contingent upon a firm’s strategic flexibility and ability to adapt. External partner collaborations, facilitated by technology, allow a firm to survive and thrive against competitive threats. External knowledge sourcing must also be linked effectively with appropriate non-technological innovations to be successful, like organization and marketing innovation. External knowledge sourcing leads to marketing innovation results when external knowledge sourcing is accompanied by appropriate organization innovation. Marketing innovation induces product innovation and enhances product innovation which subsequently enhances market performance.

A few limitations associated with this study exists which presents opportunities that future studies can address. A relatively modest amount of variance was explained by the study’s research model, which suggests that additional factors exist that enhance product innovation and market performance. External factors like abrupt changes in economic conditions, competitive environments, customer demands, or variety of managerial problems may make accurate predictions difficult.

The study findings may have been influenced by the number of highly diverse industries represented in the data sampling for a single sector–Korean manufacturers. A rigorous and comprehensive study was conducted, but industry diversity may generate too much noise to confirm the broad range of theoretical contingencies examined in this study [74]. Respondents from additional countries, cultures, and sectors might produce different results. Future research should extend the study to include respondents who are representative of additional contexts. For example, the service sector is different from the manufacturing sector and may yield intriguing insights about the interaction between external collaboration and non-technological innovation as well as external collaboration’s influence on market performance.

Future research should also examine how external knowledge sourcing is impacted by a firm’s absorptive capacity, which was outside the scope of this study. Absorptive capacity defines the amount of external knowledge is able to absorb and will likely vary from one firm to another. Future research which accounts for a firm’s absorptive capacity may yield rich findings having implications for both researchers and practitioners.
